# Buildup of Multi-Ionic Supramolecular Network Facilitated by In-Situ Intercalated Organic Montmorillonite in 1,2-Polybutadiene

**DOI:** 10.3390/polym11030492

**Published:** 2019-03-13

**Authors:** Jinhui Liu, Di Li, Xiangshuai Zhao, Jieting Geng, Jing Hua, Xin Wang

**Affiliations:** Key Laboratory of Rubber-Plastics Ministry of Education/Shandong Provincial Key Laboratory of Rubber-Plastics, College of Polymer Science and Engineering, Qingdao University of Science and Technology, Qingdao 266042, China; liujinhui7@163.com (J.L.); m13061211032@163.com (D.L.); 15192743117@163.com (X.Z.); 13475862646@163.com (J.G.)

**Keywords:** sacrificial bond, ionic network, organic montmorillonite, 1,2-polybutadiene, in-situ intercalation

## Abstract

The development of a sacrificial bond provided unique inspiration for the design of advanced elastomers with excellent mechanical properties, but it is still a huge challenge to construct a homogenous polar sacrificial network in a nonpolar elastomer. In this effort, we proposed a novel strategy to engineer a multi-ionic network into a covalently cross-linked 1,2-polybutadiene (1,2-PB) facilitated by in-situ intercalated organic montmorillonite (OMMT) without phase separation. XRD, SEM, and TEM analysis were carried out to characterize the microstructure of the resulting polymers. Crosslinking density, dielectric performance, and cyclic tensile tests were used to demonstrate the interaction of zinc methacrylate (ZDMA) and OMMT. The dynamic nature of ionic bonds allowed it to rupture and reform to dissipate energy efficiently. Stretching orientation brought parallelism between polymer chains and OMMT layers which was beneficial for the reconstruction of the ionic network, ultimately resulting in high strength and a low stress relaxation rate. Overall, our work presented the design of a uniform and strong sacrificial network in the nano-clay/elastomer nanocomposite with outstanding mechanical performances under both static and dynamic conditions.

## 1. Introduction

The nacreous layer in biological materials—byssus in mollusks and bones in mammals—provide a paradigm for a stiff, strong, and at the same time tough protective engineering [1,2]. The excellent performance of biological materials originates from a brick-and-mortar architecture, in which 95 wt % hard aragonite tablets (brick) are laminated by 5 wt % soft biopolymer matrix (mortar). Inspired by biological materials, a tremendous amount of effort has been focused on enhancing the mechanical properties of synthetic polymers by dispersing a small amount of reinforcing nano-clay into a polymer matrix [3,4,5]. Despite the undoubted success, it has remained extraordinarily challenging to obtain synergetic improvements of strength and toughness through these approaches because the biopolymer is thought to hold the key to the extraordinary toughness in biomaterials to dissipate energy as a kind of sacrificial bond [6,7,8]. Sacrificial bonds are defined as bonds that rupture before primary bonds under deformation to protect the integrity of the primary network [9]. Sacrificial bonds include irreversible associations, mainly covalent bonds [10] and reversible associations such as hydrogen bonds [11,12,13,14], metal–ligand coordinated interactions [15,16,17,18], ionic interactions [19,20], electrostatic interactions [21,22], hydrophobic associations [23], and π–π stacking [24,25]. Such reversible associations can break and reform to dissipate energy repeatedly. To combine the reinforcing effect of nano-clay and sacrificial bond, Martikainen et al. [26] prepared biomimetic nanocomposites composed of anionic nano-clay (MMT) and cationic polymer (PDDA), and then modified with dGMP to form multiple hydrogen bonds. Afterward, MMT was as brick moiety and multiple hydrogen bonds as well as electrostatic bonds were as mortar moiety, to synergistically improve the mechanical performance of the biomimetic nanocomposites.

Atactic 1,2-polybutadiene (1,2-PB) rubber with dense vinyl groups and few double bonds on backbone, exhibiting excellent wet-skid resistance, low rolling resistance, low heat build-up, and superior aging resistance, can meet the requirements of high-performance green tire when used in combination with other rubbers such as natural rubber [27,28,29,30,31,32]. Molybdenum (Mo)-based catalyst system can provide a higher activity for the coordination polymerization of 1,2-PB compared to other catalyst systems such as titanium and chromium-based catalyst system [33]. Nevertheless, the mechanical properties of 1,2-PB are expected to meet various application requirements. If 1,2-PB was reinforced by the combination of nano-clay and sacrificial bonds, it could endow 1,2-PB with unique properties. However, nano-clay and reversible sacrificial bonds are high polar motifs, whereas 1,2-PB is typically nonpolar motifs. The primary challenge to incorporate both sacrificial bonds and nano-clay into elastomers is to achieve effective dispersion. We recently reported an exfoliated nano-clay/rubber nanocomposite by an in-situ polymerization method [34,35]. The in-situ intercalated nano-clay was well dispersed in the rubber matrix with a layer-by-layer structure. OMMT, as an organic modified nano-clay, is composed of a nano-thickness silicate layer with negative charges on its surface. If there are positive charges on the molecular chain, it will interact with OMMT by ionic interactions, thereby enhancing the mechanical properties of 1,2-PB theoretically. Moreover, it has been proven that Zinc dimethacrylate (ZDMA) containing massive -(COO)_2_Zn ion pairs can graft onto rubber molecular chains through a peroxide-induced vulcanization system [36,37].

In this work, we incorporated ZDMA-induced ionic sacrificial bond into an in-situ intercalated OMMT/1,2-PB nanocomposite (in-situ-NC) to reinforce the covalently cross-linked elastomer by the interaction of ionic bond and nano-clay. The OMMT layers mostly exfoliated by in-situ polymerization served as bricks and the ionic bonds which could rupture before covalent bond acted as mortar to enhance the strength of the resulting polymer. As expected, such reinforcing engineering brought excellent properties to 1,2-PB.

## 2. Experimental Section

### 2.1. Materials

1,3-Butadiene (Bd) and n-hexane were offered by Qilu Petrochemical Corp., Zibo, China. 1,3-Butadiene was dried over activated aluminum oxide and distilled twice prior to use. n-Hexane was distilled with 67–70 °C fraction for later use. Na-MMT with the cation exchange capacity (CEC) of 60~70 mmol per 100 g was supplied by Zhejiang Fenghong Clay Chemical Co., Huzhou, China. Cetyl trimethyl ammonium bromide (CTAB), silver nitrate (AgNO_3_), ethyl alcohol, and hydrochloric acid were purchased from Tianjin Bodi Chemical Co., Ltd., Tianjin, China. The main catalyst (Mo) and co-catalyst (Al) for coordination polymerization were prepared as reported in our previous work [38]. Nitrogen was supplied by Qingdao Heli Chemical Factory, Qingdao, China. Zinc dimethacrylate (ZDMA) was purchased from J&K China Chemical Ltd., Beijing, China. Dicumyl peroxide (DCP) was used as the initiator and was supplied by Shanghai Shanpu chemical Co., Ltd.,Shanghai, China.

### 2.2. Preparation of OMMT

MMT (20 g) was dispersed into 1 L deionized water, heated to 80 °C and stirred for 2 h. Thereafter 18 mmol of CTAB in water solution was slowly added to the MMT suspension and stirred for 3 h with the PH value being adjusted to 3 to 5. After the reaction was finished, the suspension was filtered with deionized water, until there was no bromide ion detected by AgNO_3_ with a concentration of 0.1 M. Then the product was dried under vacuum at 80 °C to constant weight and ground to powder.

### 2.3. Preparation of In-Situ-NC

Under a nitrogen atmosphere, a certain amount of OMMT and Bd solution in n-hexane (with a concentration of 2.6 M) were firstly added into a reactor and heated to 60 °C. Then, the main catalyst (Mo/Bd by a molar ratio of 2 × 10^−3^) and co-catalyst (Al/Mo by a molar ratio of 30) were subsequently injected into the reactor to react for 6 h. Then the polymerization was terminated by ethyl alcohol. After the termination of the polymerization, the resulting nanocomposite was ultimately poured into the boiling water to precipitate and remove most of the main catalyst and co-catalyst. Then the precipitates were dried in vacuum at 80 °C to a constant weight. The resulting in-situ intercalated OMMT/1,2-PB nanocomposite was named as in-situ-NC.

By contrast, 1,2-PB was fabricated via the polymerization process mentioned above but without OMMT, and then the resulting polymer was physically blended with OMMT (wt % being of 4 in Bd). The resulting physical blended OMMT/1,2-PB nanocomposite was named as ph-NC.

### 2.4. Preparation of In-Situ-NC/ZDMA

In-situ-NC or ph-NC was compounded with ZDMA in a two-roll mill, respectively. Then the samples were subjected to compression molding at 135 °C. The formulations are listed in Table S1. The resultants were coded as in-situ-NC-x and ph-NC-x, respectively, where x was the mass fraction of ZDMA to NC.

### 2.5. Characterization

#### 2.5.1. X-ray Diffraction (XRD)

XRD patterns were carried out using a Rigaku D/max RB X-ray diffractometer (Tokyo, Japan) with the diffraction angles from 1° to 10°. CuK_α_ (λ = 0.154 nm) was used as an X-ray source with a generator voltage of 40 kV and a current of 100 mA.

#### 2.5.2. Gel Permeation Chromatography (GPC)

The molecular weight and molecular weight distribution of 1,2-PB and the in-situ-NC were characterized with a Waters Co. Maxims 1515 GPC instrument (Milford, MA, USA). The GPC measurements were conducted by injecting 100 μL sample solution at a flow rate of 1.0 mL/min at room temperature with tetrahydrofuran as the leaching solvent. The specimen concentration was 1 mg/mL.

#### 2.5.3. Fourier Transform Infrared Spectroscopy (FTIR)

FTIR was obtained on a Nicolet FTIR-Magna-750 spectrophotometer (Thermo Nicolet Corporation, Madison, WI, USA) in the range of 4000–400 cm^−1^ by total reflection mode.

#### 2.5.4. ^1^H Spectra of Nuclear Magnetic Resonance (^1^H NMR)

^1^H spectra of NMR were measured on a Bruker Ultra-Shield™ spectrometer (Karlsruhe, Germany) at 500 MHz with deuterated chloroform as the solvent and tetramethylsilane as an internal chemical shift reference.

#### 2.5.5. Scanning Electron Microscopy (SEM)

SEM analysis was performed on a JMS-6700F (JEOL, Mitaka, Japan) scanning microscope equipped with an energy-dispersive X-ray (EDX ISIS 300, Oxford, UK) microanalytical system. The samples were cryo-fractured in liquid nitrogen prior to SEM testing.

#### 2.5.6. Energy Dispersive Spectroscopy (EDS)

The EDS spectra and EDS maps were carried out by energy dispersion spectroscopy (E-max, Hitachi, Tokyo, Japan).

#### 2.5.7. Transmission Electron Microscopy (TEM)

For the observation of transmission electron microscopy (TEM), the specimens were ultramicrotomed into thin pieces of ~100 nm in thickness with UC7-532319 (Leica, Solms, Germany) microtome under a liquid nitrogen atmosphere. Then, the observations were carried out on a JEM-3010 (UHR, JEOL, Japan) transmission electron microscope at an acceleration voltage of 100 KV.

#### 2.5.8. Crosslinking Density

Samples were immersed in toluene at room temperature for 72 h to achieve their swollen equilibrium. Then, the swollen samples were blotted with tissue paper to remove the excess toluene and immediately weighed. Finally, the samples were dried at 80 °C to constant weight. The crosslinking density was determined from the Flory–Rehner equation [39]:

(1) $$-\left[\ln\left(1-\Phi_{r}\right)+\Phi_{r}+\chi \Phi_{r}^{2}\right]=V_{0}n\left(\Phi_{r}^{\frac{1}{3}}-\Phi_{r}/2\right)$$

where *Φ_r_* is the volume fraction of the rubber in the swollen mass, *V*_0_ is the molar volume of the toluene which is 106.2 cm^3^, *n* is the crosslinking density, and *χ* is the Flory-Huggins polymer-solvent interaction term, which is 0.393 for toluene [40]. The *Φ_r_* was obtained according to Bala et al. [41]

(2) $$\Phi_{r}=\frac{m_{2}/\rho_{2}}{m_{2}/\rho_{2}+\left(m_{1}-m_{2}\right)/\rho_{1}}$$

where *m*_1_ and *m*_2_ are the masses of the swollen sample before and after dried, respectively. *ρ*_1_ and *ρ*_2_ are the densities of toluene (*ρ*_1_ = 0.865 g/cm^3^) and rubber, respectively.

#### 2.5.9. Differential Scanning Calorimetry (DSC)

Glass transition temperature (*T*_g_) was conducted using a TA DSC-Q20 thermal analyzer, Milford, MA, USA, over a temperature range from −80 to 40 °C at a heating rate of 10 °C/min.

#### 2.5.10. Dynamic Mechanical Properties (DMA)

Dynamic mechanical properties were performed by DMA Q800 (TA, Milford, MA, USA) under temperature sweeping mode from −60 to 80 °C at a heating rate of 3 °C/min, an amplitude of 10 µm and a frequency of 1 Hz.

#### 2.5.11. Stress-Relaxation Experiment

Under different temperatures (40, 60, 80, and 100 °C), samples were quickly stretched to 50% and maintained for 10 min. Then the stress was reduced to zero, and the residual deformation was measured for 10 min.

#### 2.5.12. Hysteresis Loss

The loading-unloading cycles were performed on a DMA Q800 (TA, Milford, MA USA) instrument with an extension rate of 50%/min at room temperature. In each cycle, the sample was stretched to 100% strain. After the second cycle, the sample was heated at 80 °C for 10 min and then cooled at 25 °C for 10 min to heal the dynamic network, followed by another loading–unloading cycle.

#### 2.5.13. Dielectric Performance

Dielectric performance was performed on an Alpha-A (Novocontrol Technologies, Montabaur, Germany) novocontrol under frequency sweeping mode from 10^7^ to 10^−2^ Hz with a voltage of 1 V at room temperature. The samples were subjected to gold sputtering for 100 s before testing.

#### 2.5.14. Physical and Mechanical Properties

Tensile and tear strength were tested on a GT-AT-7000M (Taiwan Gotech, Taiwan, China) electronic tension testing machine at a speed of 500 mm/min. Hardness test was performed on a shore A hardness test instrument according to ISO 7619-1:2010. DIN abrasion test was performed on a GT-7012-D abrasion testing machine according to ISO 4649-2010.

## 3. Results and Discussion

### 3.1. In-Situ Intercalation of OMMT

**Figure 1:**
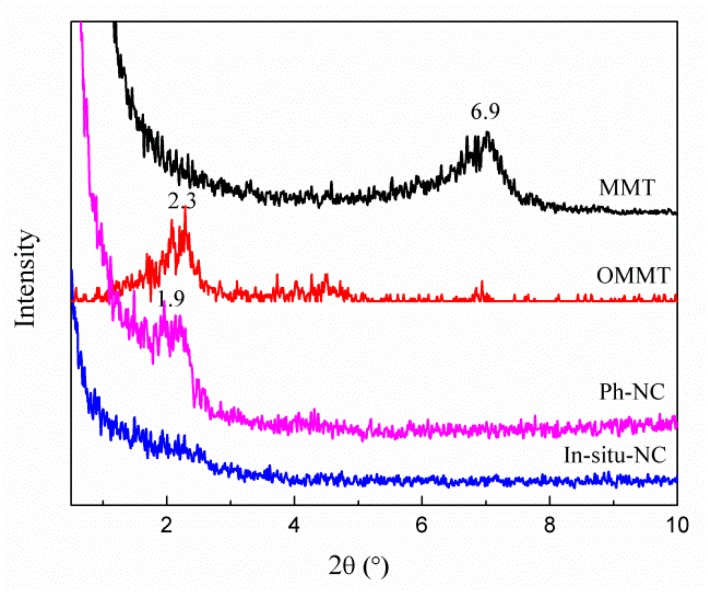
X-ray diffraction patterns of montmorillonite (MMT), organic MMT and 1,2-PB nanocomposite (ph-NC), and in-situ-NC.

Organically modified montmorillonite (OMMT) with lower surface energy and larger interplanar spacing exhibits better dispersity in organic materials than pure montmorillonite (Na-MMT). XRD patterns of Na-MMT and OMMT were shown in Figure 1. The diffraction angle of Na-MMT was 6.9° corresponding to an interlayer spacing of 1.24 nm according to Bragg’s equation (2*dsinθ* = *n λ*). After the organic modification, the diffraction angle of OMMT was 2.21°, indicating that the interlayer spacing was enlarged to 4.01 nm. Then the OMMT was in-situ intercalated with Bd via Mo-catalyzed coordination polymerization. The relationship between the conversion rate of Bd monomer and the mass fraction of OMMT was shown in Figure S1 (Supplementary Information). With the increasing mass fraction of OMMT, the conversion rate of Bd decreased and became less than 30% when the OMMT was more than 6 wt %, because the anions on OMMT would deactivate the Mo-catalyst. To ensure a relative higher conversion rate and more intercalated structure, the mass fraction of OMMT for the following experiments was settled as 4%. Subsequently, the resulting in-situ OMMT/1,2-PB nanocomposite (in-situ-NC) was subjected to XRD analysis. The OMMT in the in-situ-NC showed no peak, which indicated that OMMT was mostly exfoliated as individual OMMT layers in the 1,2-PB matrix. Some small OMMT aggregates still existed in in-situ-NC (as shown by TEM images) but showed no peak. That was attributed to the fact that the amount of the OMMT aggregates and the number of the layers constituting these aggregates were low. Consequently, the small aggregates might be embedded by the rubber matrix and were difficult to detect. By contrast, the physical blended OMMT/1,2-PB nanocomposite (ph-NC) showed a peak at 2.1°, demonstrating that the OMMT was stacked.

The degree of polymerization and molecular weight distribution were determined by gel permeation chromatography (GPC, Figure S2). The degree of polymerization of 1,2-PB and in situ NC was 12,777 and 16,703, respectively. For in-situ-NC, the degree of polymerization is larger, because the polymerization is conducted among OMMT layers, in which partial chain transfer reaction was inhibited. Therefore, the life of the active center was prolonged and ultimately resulted in higher molecular weight. The molecular weight distribution of 1,2-PB and in-situ-NC was 2.91 and 2.76, respectively, demonstrating that the OMMT had little effect on it.

**Figure 2:**
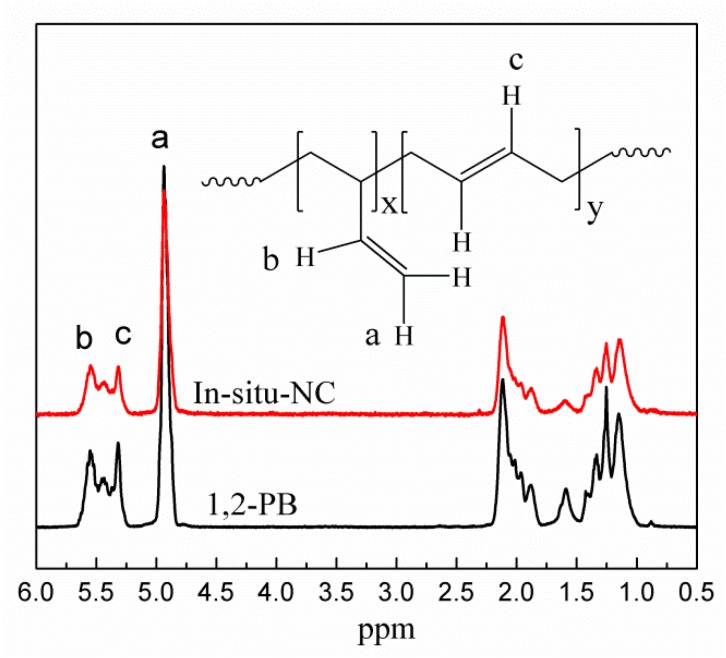
^1^H Nuclear Magnetic Resonance (NMR) spectra of 1,2-PB and in-situ-NC.

The 1,2-unit content of 1,2-PB and in-situ-NC was calculated by NMR spectra (Figure 2). The signals at *δ* = 4.9, *δ* = 5.3 and *δ* = 5.5 ppm were attributed to the olefinic protons of =CH_2_ of 1,2-unit, -CH= of 1,4-unit and -CH= of 1,2-unit, respectively. The 1,2-unit content (C) was calculated according to the following equation:

(3) $$C=\frac{A_{\delta =4.9}}{A_{\delta =4.9}+\left(A_{\delta =5.3\sim 5.5}-1/2\times A_{\delta =4.9}\right)}\times 100\%$$

where *A* was the integral area of the corresponding signal peak. After in-situ intercalated with OMMT, the 1,2-unit content of the NC was 83% which was the same as that of unmodified 1,2-PB. The results of FTIR and ^1^H NMR showed that, although the negative ions on the surface of OMMT may devitalize the Mo catalyst, it couldn’t change the structure of active centers in this coordinative polymerization procedure.

### 3.2. Construction of Multi-Ionic Supramolecular Network

**Scheme 1:**
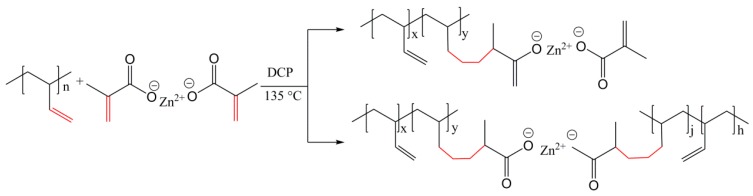
Reaction between 1,2-PB and zinc methacrylate (ZDMA).

**Scheme 2:**
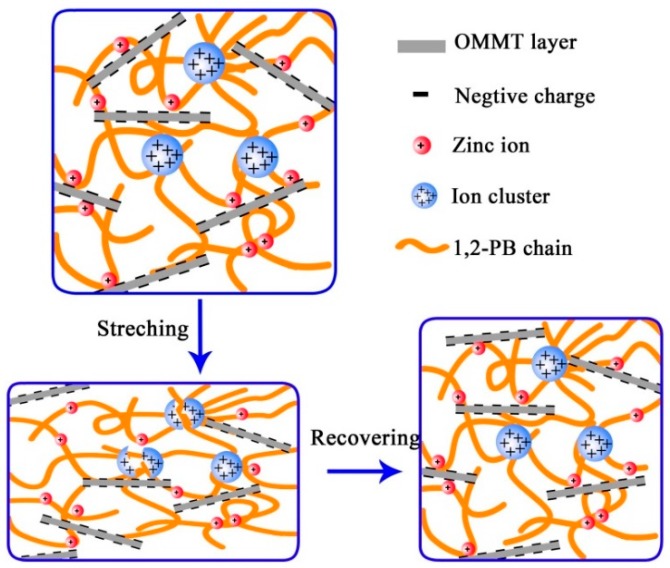
Proposed mechanism of the rupture and reconstruction of sacrificial bonds in the multi-network system of 1,2-PB.

For chemically cross-linked elastomers, a weak or transient network could be an effective strategy to endow them with enhanced mechanical properties and unexpected functionality. The weak or transient junctions can sustain and sacrifice an initial load and then preferentially break before the rupture of the covalent network of elastomers. In this effort, ZDMA was employed to construct sacrificial bonds anchoring between the rubber skeleton and the OMMT layer. ZDMA was grafted onto the polymer molecular chains by the reaction of the double bond between ZDMA and 1,2-PB (Scheme 1) to incorporate -(COO)_2_Zn groups into rubber matrix. Then the Zinc ion (Zn^2+^) was able to joint with the negative ion on OMMT to form OMMT-polymer ionic bonding or with the negative ion on an adjacent polymer to form inter-chain and intra-chain ionic bonding. Additionally, -(COO)_2_Zn groups could aggregate together to create a small ion cluster. Therefore, OMMT-polymer ionic bonding, inter-chain, and intra-chain ionic bonding together with ionic cluster constituted a multi-ionic supramolecular network (Scheme 2). During stretching, the multi-ionic network would rupture before the covalent network and then was reconstructed after unloading.

**Figure 3:**
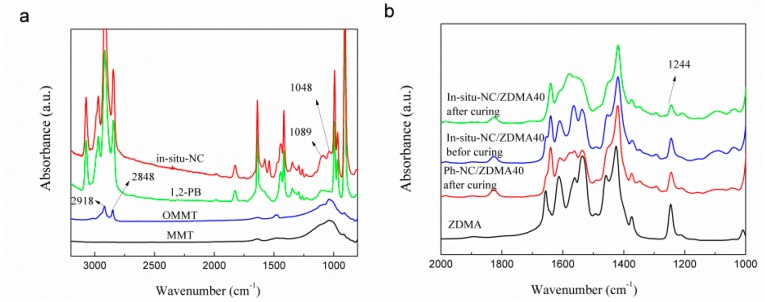
FTIR spectra of (**a**) MMT, OMMT, 1,2-PB, and in-situ-NC, (**b**) ZDMA, in-situ-NC/ZDMA40 before and after curing, and ph-NC/ZDMA40.

The organic modification of MMT could be further verified by FTIR analysis. As shown in Figure 3a, after organic modification, new peaks appeared at 2918 and 2848 cm^−1^, respectively, which were attributed to the asymmetric stretching vibrations of methyl and methylene groups of CTAB. The absorption peaks at 1089 and 1048 cm^−1^ corresponded to the stretching vibration band of Si–O in MMT. In comparison with ph-NC/ZDMA40, the peak intensity of in-situ-NC/ZDMA40 at 1089 and 1048 cm^−1^ was stronger, resulting from OMMT tending to aggregate by the physical blending method while it was mostly exfoliated by the in-situ method.

Moreover, the exfoliated OMMT layers would leave a more exposed Si–O bond. Additionally, the formation of a covalent linkage between the ZDMA and the double bond on 1,2-PB was illustrated by FTIR analysis (Figure 3b). In the case of ZDMA, the absorption peak at 1244 cm^−1^ belongs to the vibration band of C–(C=O)–O conjugated with C=C double bonds. The relative intensity of in-situ-NC/ZDMA40, after curing, was obviously weak at 1244 cm^−1^ due to the reaction of C=C double bonds. Moreover, more ZDMA was grafted onto 1,2-PB molecular chains for in-situ-NC according to the weaker peak intensity at 1244 cm^−1^. The main reason was that the negative ions on the surface of OMMT layers led to a better dispersity of ZDMA.

**Figure 4:**
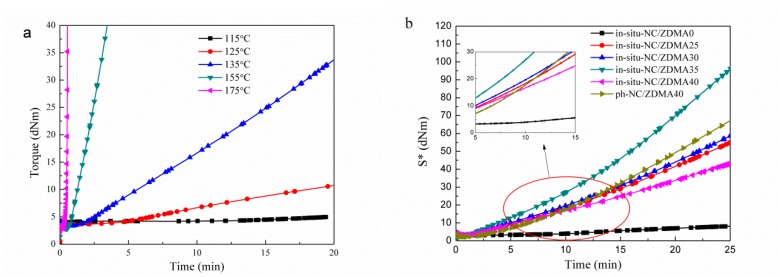
(**a**) Curing curves of in-situ-NC/OMMT40 at 115,125,135,145, and 155 °C, (**b**) curing curves of in-situ-NC/ZMDAs and ph-NC/ZDMA40 at 135 °C.

**Figure 5:**
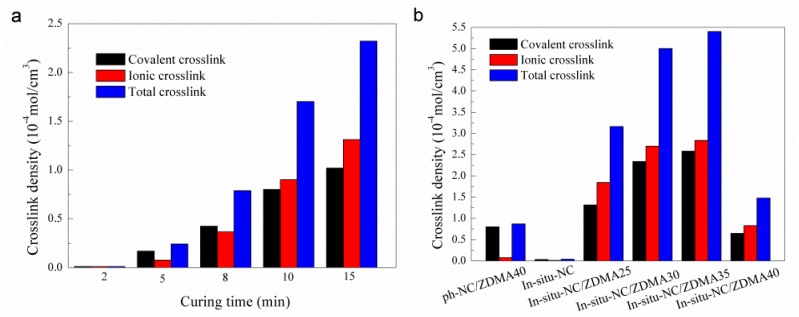
Crosslinking density of (**a**) in-situ-NC/ZDMA40 under different curing times, (**b**) Crosslinking density of in-situ-NC, in-situ-NC/ZDMAs, and ph-NC/ZDMA40.

However, the crosslinking of 1,2-PB double bonds and the grafting of ZDMA is a pair of competitive reactions. In this work, we controlled the crosslinking density of covalent and ionic networks by adjusting reaction temperature and time. The curing curves of the in-situ-NC/ZDMA40 under different temperatures were shown in Figure 4. The increased torque value is usually considered to represent the evolution of crosslinking network in rubbers [42]. With the increase in temperature, the torque rate gradually increased because of a shorter half-life period of DCP. However, at a high temperature it would be difficult to control the rate of crosslinking, and at a low temperature it would be difficult to graft ZMDA. Thus, 135 °C was selected as the curing temperature for the following experiments. Samples were swollen in toluene for 72 h to calculate the total crosslinking density. To destroy the ionic crosslinks, samples with the same size were swollen in the mixture of toluene/hydrochloric acid/ethyl alcohol for 72 h to calculate the covalent crosslinking density [36,37]. Then the ionic crosslinking density could be determined by subtraction of the covalent crosslinking density from the total cross-link density. Corresponding digital photographs of in-situ-NC/ZDMA40 cured with different times were shown in Figure S3. It is clear that the swelling volume in the mixture of toluene/hydrochloric acid/ethyl alcohol was larger than that in the toluene. This distinctly evidenced that the supramolecular network was destroyed by the hydrochloric acid, thereby resulting in larger swelling volume. The statistical evidence also demonstrated the existence of the ionic network. As shown in Figure 5a, both covalent and ionic crosslinking density increased with the increasing curing time and the ionic crosslinking density was higher than the covalent crosslinking density when the curing time exceeded 10 min. A high crosslinking density corresponds to a high tensile strength but has a low elongation at break (Figure S4). Consequently, the samples cured at 135 °C for 10 min could bring about a better reinforcing effect.

As shown in Figure 5b, the ionic crosslinking density of ph-NC/ZDMA40 was 0.07 × 10^−4^ mol/cm^3^ was significantly lower than that of in-situ-NC/ZDMAs due to the non-exfoliated OMMT. Under the same curing condition, the covalent crosslinking density for in-situ-NC was almost zero and for in-situ-NC/ZDMAs was more than 0.8 × 10^−4^ mol/cm^3^. With the increasing mass fraction of ZDMA, both ionic and covalent crosslinking densities for in-situ-NC/ZDMAs increased at first and then decreased. The increasing trend illustrated that ZDMA not only determined the formation of the ionic network but also affected the generation of covalent bond for in-situ-NC/ZDMA because the -(COO)_2_Zn groups on ZDMA might accelerate the decomposition of DCP. Therefore, the covalent crosslinking density increased significantly for ph-NC/ZDMA40 and in-situ-NC/ZDMAs. However, too much ZDMA in in-situ-NC/ZDMAs is likely to agglomerate into particles which embed DCP and thereby results in the homopolymerization of ZDMA to form poly-ZDMA (PZDMA). Accordingly, the reaction rate for the grafting of ZDMA and the crosslinking of 1,2-PB decreased for in-situ-NC/ZDMA40. Additionally, the total crosslinking density could be further confirmed by the torque value in a curing curve (Figure 4b). In short, the addition of ZMDA into the in-situ-NC could accelerate the formation of the covalent network and construct strong ionic network at the same time.

**Figure 6:**
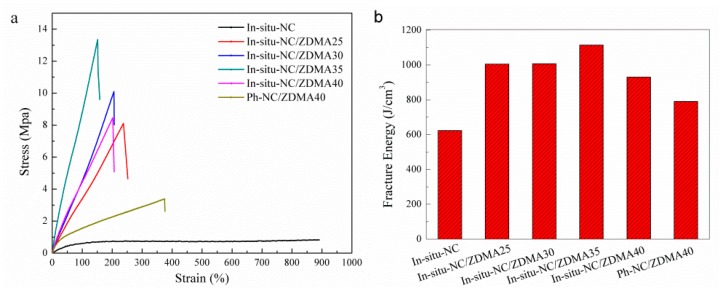
(**a**) Stress–strain curves of and (**b**) fracture energy of in-situ-NC, in-situ-NC/ZDMAs, and ph-NC/ZDMA40.

The stress–strain curves of in-situ-NC, in-situ-NC/ZDMAs, and ph-NC/ZDMA40 were presented in Figure 6a. In-situ-NC/ZDMA exhibited high breaking stress resulting from the high total crosslinking density. The reinforcing effect of the ionic network could be verified by the stress–strain curves of in-situ-NC/ZDMA40 and ph-NC/ZDMA40. Considering a similar covalent crosslink, the tensile strength of in-situ-NC/ZDMA40 was significantly higher because of higher ionic crosslinking density (Figure 6b). Nevertheless, the elongation at break of in-situ-NC/ZDMAs was relatively low which was triggered by the low mobility of the polymer chain restricted by covalent as well as ionic crosslinks.

**Figure 7:**
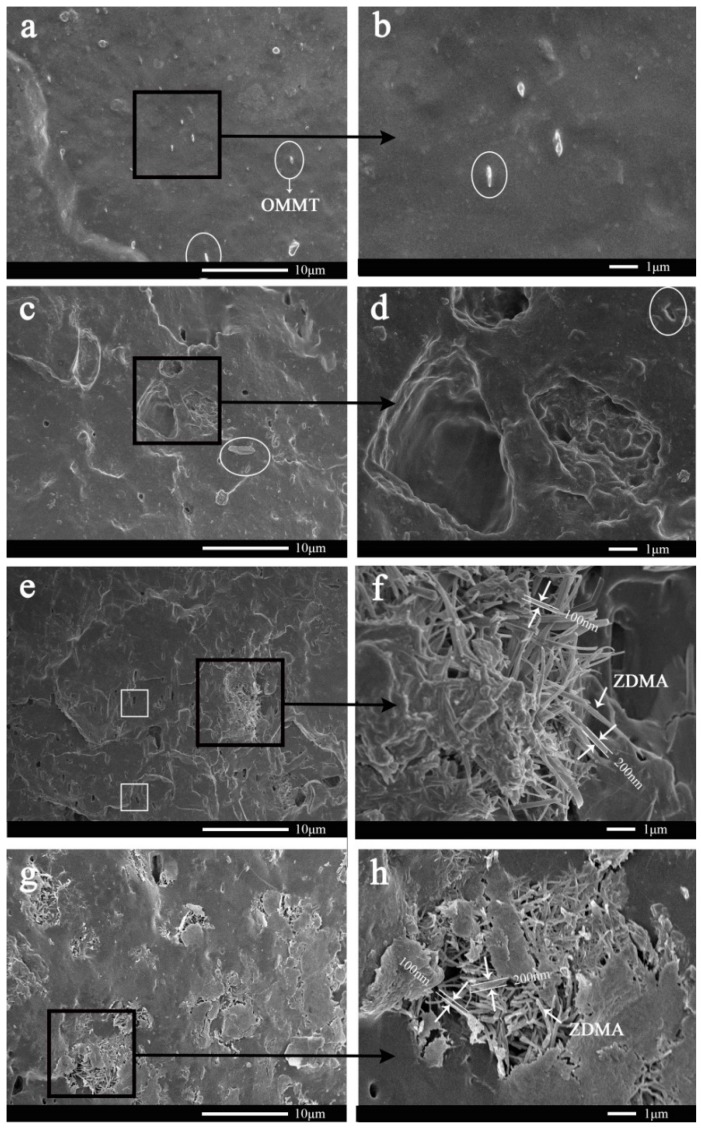
SEM images of (**a**,**b**) in-situ-NC, (**c**,**d**) in-situ-NC/ZDMA25, (**e**,**f**) in-situ-NC/ZDMA40, and (**g**,**h**) ph-NC/ZDMA40.

The dispersion morphologies of OMMT and ZDMA in 1,2-PB matrix by in-situ intercalated and physical blending methods were studied by SEM and TEM. As shown in Figure 7a, several aggregates were marked with white ellipses and some fibrils were highlighted with arrows. According to EDS spectra (Figure S5), O, K, Na, Al, and Si elements appeared in these aggregates which indicated a composition of OMMT. C, O, Zn elements appeared in those fibrils manifesting that they were composed by ZDMA. Moreover, the composition of these aggregates and fibrils were further verified by EDS maps (Figures S6 and S7), in which the distribution of C, O, Zn, and Si atoms were well consistent with the corresponding SEM image. Therefore, for the in-situ intercalated OMMT, it couldn’t be fully exfoliated leaving a few small aggregates in the polymer matrix. Such small OMMT aggregates have little effect on the polymer. For in-situ-NC/ZDMA25 (Figure 7c,d), the ZDMA was well dispersed in the 1,2-PB matrix without any agglomerated fibrils, which was attributed to the fact that the well exfoliated OMMT would facilitate the dispersion of ZDMA via the ionic interaction. However, the ZDMA began to agglomerate with each other when the fraction of ZDMA reached 40 phr (Figure 7e,f). The ZDMA in ph-NC/ZDMA40 (Figure 7g,h), by contrast, formed agglomerates more easily due to the poor dispersity of OMMT by the physical blending method.

**Figure 8:**
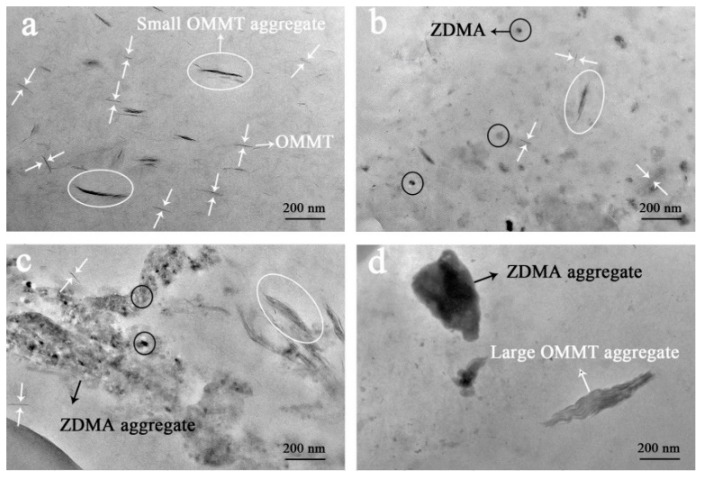
(**a**) TEM images of in-situ-NC, (**b**) in-situ-NC/ZDMA25, (**c**) in-situ-NC/ZDMA40, and (**d**) ph-NC/ZDMA40.

The TEM images were shown in Figure 8. In the case of the nanocomposites prepared by the in-situ method, OMMT in the 1,2-PB matrix (Figure 8a) was mostly exfoliated with a few small OMMT aggregates stacked by several layers. However, in the case of the nanocomposite prepared by the physical blending method, OMMT tended to be large aggregates stacked by dozens of layers (Figure 8d). ZDMA was dispersed into in-situ-NC/ZDMA25 (Figure 8b) in nanoscale without agglomerates which was consistent with the result of SEM. ZDMA aggregated to incompact lumps in in-situ-NC/ZDMA40 (Figure 8c) whereas it aggregated to compact chunks in ph-NC/ZDMA40 (Figure 8d), which was in accordance with the analysis of crosslinking density. These large agglomerates would act as impurities under loading and decrease the mechanical properties of the resulting polymer.

**Figure 9:**
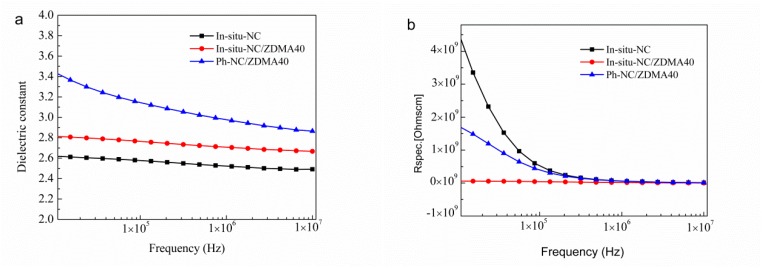
(**a**) Dielectric constant and (**b**) volume resistivity of in-situ-NC, in-situ-NC/ZDMA40, and ph-NC/ZDMA40 under the frequency sweeping mode.

The polarity of the polymer materials can be discriminated based on the dielectric constant. Generally, a polymer typically exhibits a dielectric constant of less than 10 [43]. As shown in Figure 9, with the same mass fraction of ZDMA, the dielectric constant of ph-NC/ZDMA40 was evidently higher than that of in-situ-NC/ZDMA40, illustrating that the ph-NC displayed a higher polarity. For in-situ-NC/ZDMA40, ZDMA was dispersed in 1,2-PB in nanoscale which was entirely integrated with 1,2-PB/OMMT nanocomposite. Therefore, the polarity of in-situ-NC/ZDMA40 was equivalent to the linear addition of the ZDMA and 1,2-PB/OMMT. However, in the case of ph-NC/ZDMA40, ZDMA dispersed in the 1,2-PB matrix mainly in large aggregates and was easily separated from 1,2-PB and OMMT. Afterward, ZDMA with a higher polarity had a greater effect on the polarity than the linear consideration. Nevertheless, the volume resistivity of in-situ-NC/ZDMA40 was relatively lower because the ion exchange in the ionic network helped charge transport. Consequently, the decrease in volume resistivity indicated that the ionic network could improve the antistatic property of the resulting rubber. The polarity and resistivity results indicated that ZDMA could construct a uniform ionic network in in-situ 1,2-PB/OMMT and it could conduct ion exchange.

DMA testing typically shows a small sample deformation during modulus measurements. In this way, the sample can respond to deformation by changing the chain conformation. When the deformation is accommodated by conformational changes, without bending or breaking of bonds, the relationship between rubbery plateau modulus and crosslinking density (*V*e) can be described as follows [44]:

*V*e = E′/(3*ρ*RT) (4)

where the modulus (E′) was obtained in the rubbery plateau (usually take the value of peak temperature of loss modulus plus 40 °C), T is the temperature in °K corresponding to the storage modulus value, R is the gas constant (8.314 × 10^7^ ergs/K mol), and *ρ* is the density of the polymer. Thus the total crosslinking density could be calculated by DMA and the results were shown in Figure S9. The results of the total crosslinking density calculated by DMA were a bit higher than the results calculated by equilibrium swelling method (Figure 5) but the tendency of both methods was similar.

**Figure 10:**
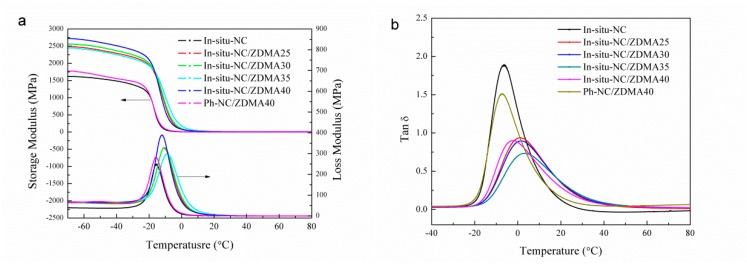
(**a**) Dependence of E′ and E″ and (**b**) tan δ versus temperature for in-situ-NC, in-situ-NC/ZDMAs and ph-NC/ZDMA40.

Figure 10a illustrated the temperature dependence of the storage modulus (E′) and loss modulus (E″) for NC/ZDMAs. E′ of in-situ-NC/ZDMAs was constantly improved with increasing ZDMA fraction because the ionic crosslinks provided a force against deformation. In-situ-NC and ph-NC/ZDMA40 showed a peak of loss modulus at around −10 °C, while that of in-situ-NC/ZDMAs were at around 0 °C. We suspected that the high crosslinking density restricted the movement of the primary network which would relax at a relatively high temperature. The effect of the ionic network could be proved by the modulus of in-situ-NC/ZDMA40 and ph-NC/ZDMA40. With the almost identical covalent network, the larger ionic network led to a higher storage modulus for in-situ-NC/ZDMA40. The peak value of loss modulus obviously increased for in-situ-NC/ZDMAs since the restricting effect of sacrificial bond on chain segment induced more chain friction during conformational motion. Therefore, the incorporation of ionic network increased the storage and loss modulus at the same time. As shown in Figure 10b, in comparison to in-situ-NC and ph-NC/ZDMA40, the peak value of tan δ for in-situ-NC/ZDMAs was significantly suppressed due to the remarkably enhanced E′ and slightly improved E″. Moreover, the temperature of tan δ peak for in-situ-NC/ZDMA40 was higher than that of ph-NC/ZDMA40 because of the high ionic crosslinking density. For in-situ-NC/ZDMAs, with the increasing mass fraction of ZDMA, the temperature of peak tan δ increased at first and then decreased which was consistent with the results of crosslinking density too. In brief, the incorporation of ionic crosslinks led to lower mobility of the polymer chain and resulted in a higher relaxing temperature for polymer chain segment.

**Figure 11:**
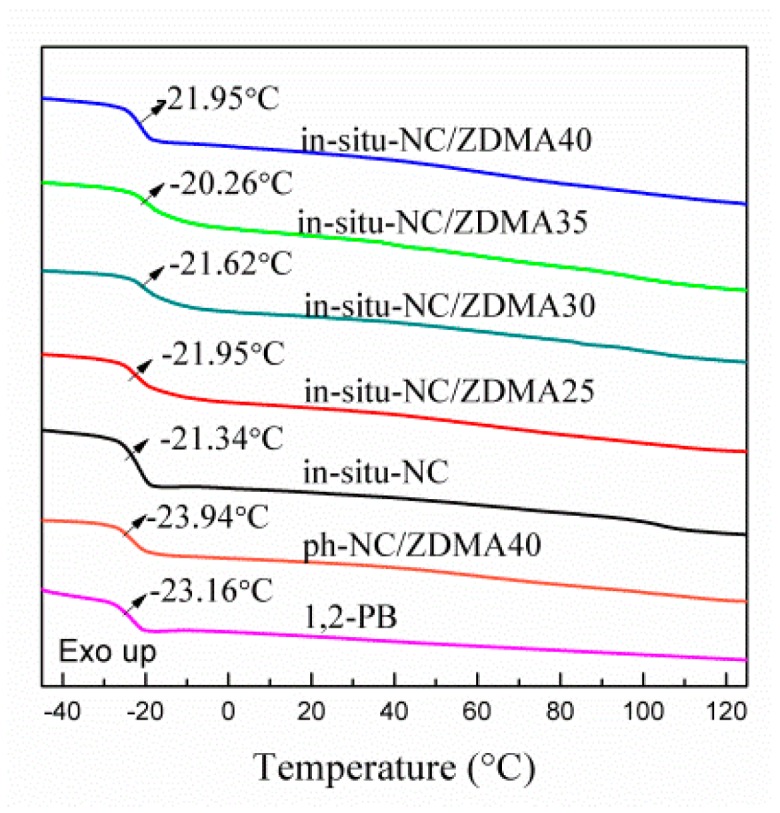
Differential Scanning Calorimetry (DSC) curves of 1,2-PB, in-situ-NC, in-situ-NC/ZDMAs, and ph-NC/ZDMA40.

Among the ionic crosslinks, large ionic clusters drastically reduce the dynamics of polymer segments around them and create layers of the trapped polymer with gradually reduced mobility. If the aggregation is significant enough to lead to phase separation, this polymer fraction would show its own glass transition (named ionic transition) [45]. According to the variation of the loss factor with temperature (Figure 10b), there is only one transition of the ionic modified polymers. Additionally, the glass transition temperature (*T*_g_) determined by DSC (Figure 11) also showed no ionic transition in in-situ-NC/ZDMAs. Therefore, the ionic cluster in this system was not strong enough to create phase-separated morphology. Moreover, the *T*_g_ of in-situ-NC was about 1.8 °C higher than that of pure 1,2-PB because of the exfoliated OMMT layers. It could be concluded from the DSC curve of ph-NC/ZDMA40 that ZDMA aggregates would enlarge the inter-chain distance and decrease the *T*_g_ of the polymer. For in-situ-NC/ZDMAs, with the increasing mass fraction of ZDMA, the *T*_g_ increased first and then decreased slightly. The increase of *T*_g_ was attributed to the fact that the ionic crosslinks could enhance the interaction among polymer chains and restrict the movement of the chain segment. Nevertheless, the aggregated ZDMA in in-situ-NC/ZDMA40 reduced the ionic crosslinking density and decreased *T*_g_ slightly.

**Figure 12:**
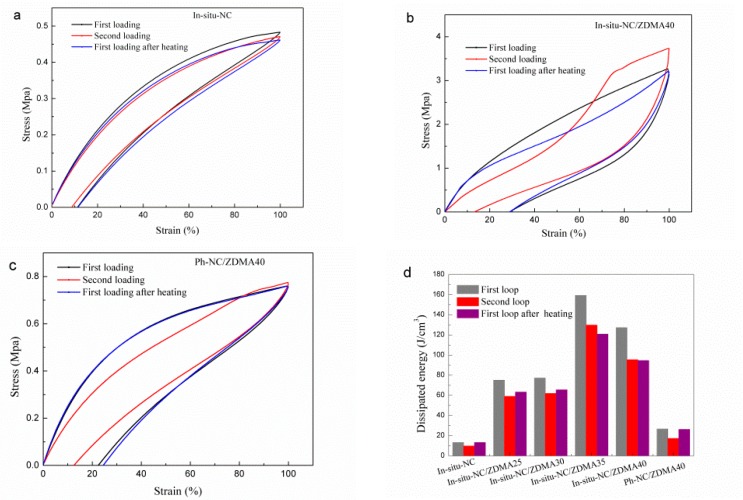
Cyclic tensile curves of (**a**) in-situ-NC, (**b**) in-situ-NC/ZDMA40 and (**c**) ph-NC/ZDMA40 and (**d**) the corresponding dissipation of energy.

Sacrificial bond is a general conception of weaker bond in polymer and it is difficult to prove its sacrifice directly. Rief et al. reported that the excellent strength and toughness in natural materials could be characterized by a saw-tooth pattern observed from single-molecule force–extension curves [46]. The saw-tooth pattern was also reported in synthesized polymers with sacrificial bonds and this pattern is a direct evidence of the sacrifice of the sacrificial bonds [17]. Meanwhile, Etienne Ducrot et al. showed that the break of the sacrificial bond could be watched by intensity-colorized images in chemiluminescent cross-linking molecules, which emit light as they break, mapped in real time [10]. However, these direct characterization methods could only be carried out by incorporating chemiluminescent molecules or using special modified AFM instrument. Fortunately, some studies reported that the sacrifice of the sacrificial bond could be verified indirectly. Baochun Gou et al. [18,47] demonstrated that the sacrifice of the sacrificial bond could be proved by hysteresis loss and stress relaxation testing. To confirm the sacrifice and reconstruction of the ionic bonds, cyclic tensile tests were performed with stretching to a predefined 100% strain and the corresponding dissipation of energy was shown in Figure 12. As expected, low hysteresis was observed for in-situ-NC (Figure 12a) and ph-NC/ZDMA40 (Figure 12c) in the first, second, and after heating cycle, which is reasonable for dominance of covalent crosslinks. As for the samples prepared by in-situ polymerization, taking in-situ-NC/ZDMA40 as an example (Figure 12b), significant hysteresis was observed in the first loading–unloading cycle on account of the effect of the ionic network. The dissipation of energy during the loading–unloading test was defined as the area surrounded by the circle and was shown in Figure 12d. The improved dissipation of energy for in-situ-NC/ZDMAs in the first loading–unloading cycle was associated with the rupture of the ionic network. For the second loading, the stress at 100% strain of in-situ-NC/ZDMA40 was higher than that of the first loading which doesn’t exist for in-situ-NC and ph-NC/ZDMA40. We speculated that the stretching orientation of polymer chains and OMMT layers remained after the first unloading due to its slower relaxation rate indicated by *T*_g_. The orientation of OMMT layers and polymer chains were in the same direction, which was advantaged for the reconstruction of the ionic network. Meanwhile, the increase in the maximum stress displayed the formation of a denser ionic network induced by the orientation mentioned above. After heating at 80 °C, the orientation of the covalent network of in-situ-NC and ph-NC/ZDMA40 nearly completed self-recovery, and its cyclic tensile curve almost overlapped that of the first cycle. However, in-situ-NC/ZDMA40 showed smaller stress during loading after heating than that of the first cycle before heating. It seems to form a weaker ionic network after heating, which, combining with the denser network by stretching, indicated that the ionic network is sensitive to the conformation of polymer chains. In addition, the samples with other content of ZDMA exhibited similar hysteresis behaviors (Figure S8).

**Figure 13:**
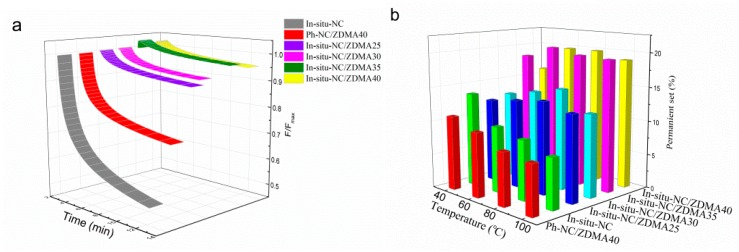
(**a**) Stress relaxation curves and (**b**) residual deformation after recovery for 10 min under respective temperatures.

To further illustrate the reinforcing effect of ionic crosslinks, stress relaxation analysis, was performed at 25 °C (Figure 13a). All the samples were quickly subjected to a strain of 50%, and then the strain was maintained for 600 s. Compared with in-situ-NC and ph-NC/ZDMA40, in-situ-NC/ZDMAs released the applied force much slowly, which implied the protection of ionic bonds to the primary network under loading. This relaxing rate was contrary to the result of polymers reinforced by hydrogen bonds and coordination bonds. Baochun Gou et al. [18,47] manifested that the hydrogen bonds and coordination bonds could disassociate under stretching and the relaxing rate was much more rapid than that of the polymer without a sacrificial network. In this effort, the slow stress relaxation rate of in-situ-NC/ZDMAs might result from the reconstruction of the ionic network facilitated by stretching orientation. The stress of in-situ-NC/ZDMA, especially for in-situ-NC/ZDMA35, was a bit higher in the early stage than that of the initial stress, further demonstrating the reconstruction of the ionic network.

After the relaxation experiment and recovery for 10 min, the residual deformation was measured and was shown in Figure 13b. The residual deformation of in-situ-NC/ZDMA35 was significantly larger than that of the others because of the highest ionic crosslinking density. The ionic crosslinks would break and recombine with the adjacent ions and finally prevent the recovery of the deformed covalent bond. Nevertheless, in-situ-NC/ZDMA40 exhibited higher residual deformation than in-situ-NC/ZDMA25 and 30, which was induced by a relatively low ionic crosslinking density due to PZDMA with low molecular weight. Additionally, as the testing temperature increased, the residual deformation of in-situ-NC and ph-NC/ZDMA40 continually decreased whereas it increased first and then decreased for in-situ-NC/ZDMAs. The deformation of ph-NC/ZDMA40 and in-situ-NC was dominated by the covalent network. At elevated temperatures, the chain segment mobility was improved which resulted in a stronger contractive force, ultimately limiting the residual deformation. However, the deformation of in-situ-NC/ZDMAs was determined by both the covalent and ionic network. Under a quick stretching condition, the partial ionic network was quickly destroyed and was unable to be in-situ reconstructed. Subsequently, the fractured ionic crosslinks reunited with adjacent ions and the reuniting rate increased at elevated temperature, thereby leading to a larger residual deformation. Synchronously, the mobility of the chain segment was improved resulting in a higher contractive force. Therefore, the residual deformation decreased slightly at 100 °C. Overall, the reversible ionic network endowed the polymer with a more plastic property which meant it may be used as a shape memory material.

The mechanical properties of in-situ-NC, in-situ-NC/ZDMAs and ph-NC/ZDMA40 were listed in Table 1. The tensile strength and tear strength of in-situ-NC/ZMDAs were obviously higher than that of ph-NC/ZDMA40 on account of the ionic crosslinks formed between the exfoliated OMMT and the -(COO)_2_Zn groups. Nevertheless, the mechanical properties of in-situ-NC/ZDMA40 decreased attributed to the large ZDMA aggregates acting as impurities. Meanwhile, the abrasion resistance of in-situ-NC and in-situ-NC/ZDMAs decreased with the increasing fraction of ZDMA owing to the fact that the ionic contacts decreased the flexibility of the polymer chains. Moreover, the abrasion loss of ph-NC/ZDMA40 was also very high, resulting from the weak interaction of the polymer chain and ZDMA aggregates.

## 4. Conclusions

We developed a nano-clay/elastomer nanocomposite with high tensile strength, tearing strength and low stress relaxation rate which was reinforced by a multi-ionic supramolecular network including OMMT-polymer ionic crosslink, inter-chain, or intra-chain ionic crosslink as well as small ion cluster. The exfoliated OMMT layers prevented the aggregation of ZDMA in nonpolar rubber matrix through ionic interaction between OMMT and ZDMA, and thereby resulted in a uniform ionic network. The ionic network could rupture preferentially during stretching to protect the covalent network and dissipate more energy. Additionally, the ionic crosslinks could be reconstructed and be strengthened through the orientation of OMMT and polymer chain. However, the reversibility of such ionic bonds enlarged the residual deformation of the resulting polymer. In future studies, we will further improve the ionic crosslinking density and construct a stronger sacrificial network to prepare shape memory or self-recovery materials and study the dynamics of ionic crosslinking.

## Figures and Tables

**Table 1:** Mechanical properties of in-situ-NC, in-situ-NC/ZDMAs, and ph-NC/ZDMA40.

Sample	ph-NC/ZDMA40	In-Situ-NC	In-Situ-NC/ZDMA25	In-Situ-NC/ZDMA30	In-Situ-NC/ZDMA35	In-Situ-NC/ZDMA40
Tensile strength (Mpa)	2.82	0.99	7.98	8.10	11.40	8.45
Tensile stress at 100% elongation (MPa)	1.14	0.60	4.51	3.37	5.82	4.24
Elongation at break (%)	375	800	205	237	213	202
Tearing strength (kN/m)	28.05	11.87	23.28	25.74	31.23	29.48
Shore A hardness (°)	67	45	59	61	66	65
Abrasion loss (cm^3^·1.6 km^−1^)	0.38	0.28	0.33	0.34	0.35	0.37

## Supplementary Materials

The supplementary materials are available online at https://www.mdpi.com/2073-4360/11/3/492/s1.
