# Supplementary material for: Buildup of Multi-Ionic Supramolecular Network Facilitated by In-Situ Intercalated Organic Montmorillonite in 1,2-Polybutadiene
Source: Polymers (Basel). 2019 Mar 13;11(3):492. doi: 10.3390/polym11030492 (PMC6473310; doi:10.3390/polym11030492)
Supplement: Supplementary file 1 [file polymers-11-00492-s001.pdf]

Supporting Information

Table S1 Formulations of ph-NC/ZDMA40, in-situ-NC and in-situ-NC/ZDMAs

Samples	Mass fraction of OMMT(%)	Mass fraction of ZDMA（%）	Mass fraction of ZDMA (%)
ph-NC/ZDMA40	4	40	1
In-situ-NC	4	0	1
In-situ-NC/ZDMA25	4	25	1
In-situ-NC/ZDMA30	4	30	1
In-situ-NC/ZDMA35	4	35	1
In-situ-NC/ZDMA40	4	40	1

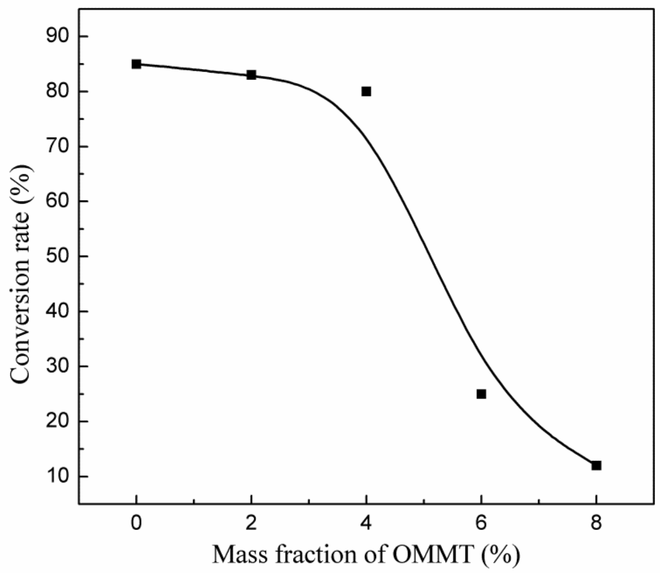

Figure S1 Conversion rate of Bd with different mass fraction of OMMT

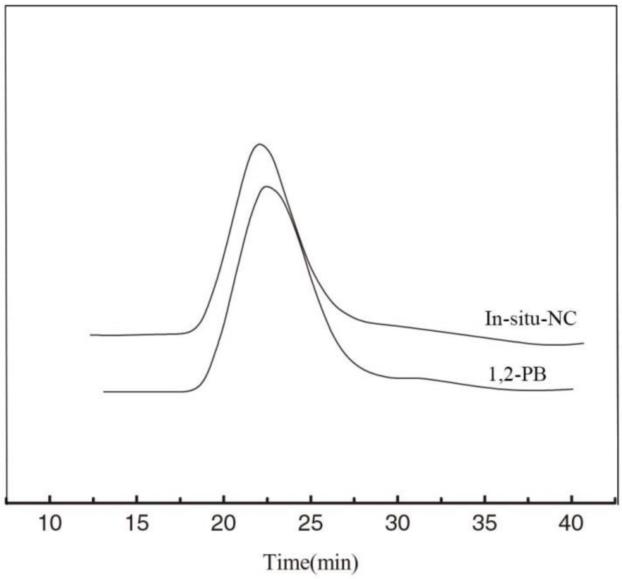

Figure S2 GPC curves of 1,2-PB and in-situ-NC

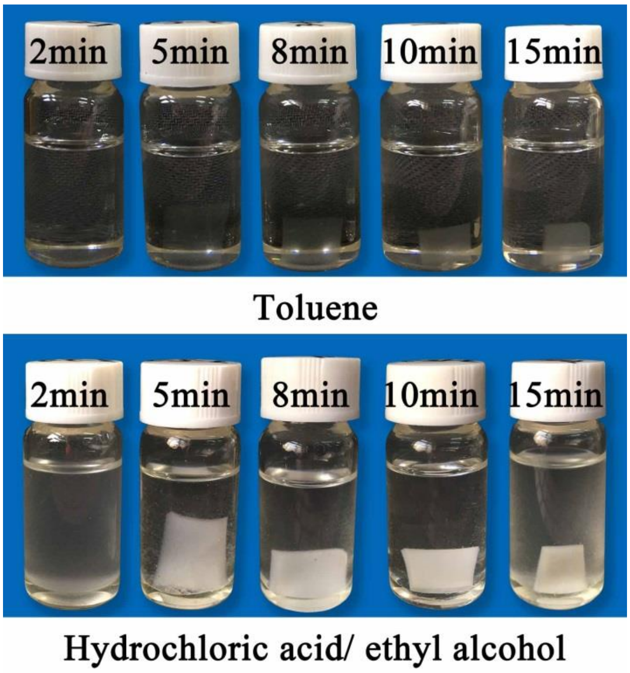

Figure S3 Digital photographs of in-situ-NC/ZDMA40 cured for different times

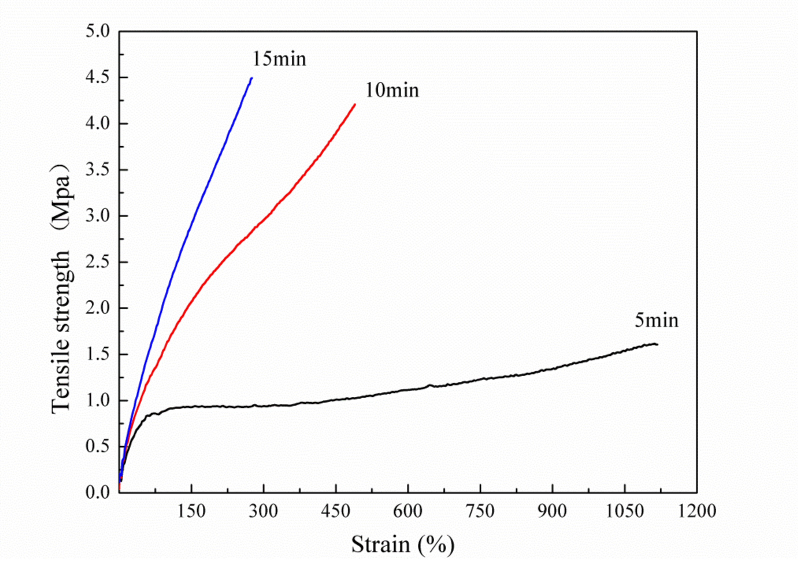

Figure S4 Stress-strain curves of in-situ-NC/ZDMA40 with the curing time of 5,10 and 15 min at 135℃

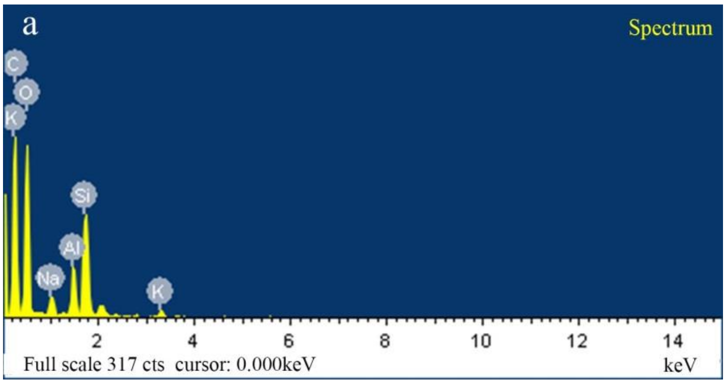

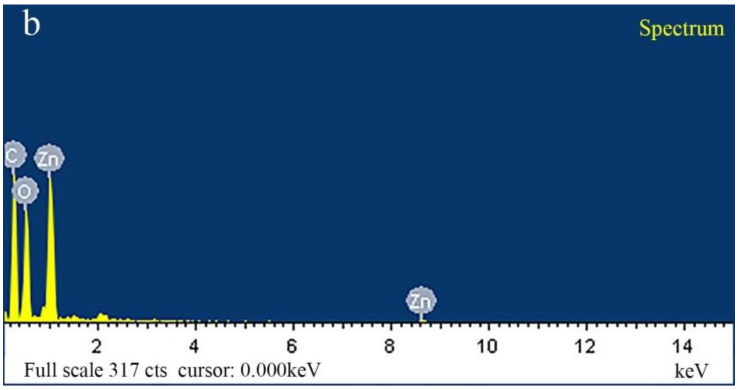

Figure S5 EDS spectra of (a) aggregate marked with white ellipse and (b) fibrils marked with arrows in Figure 7

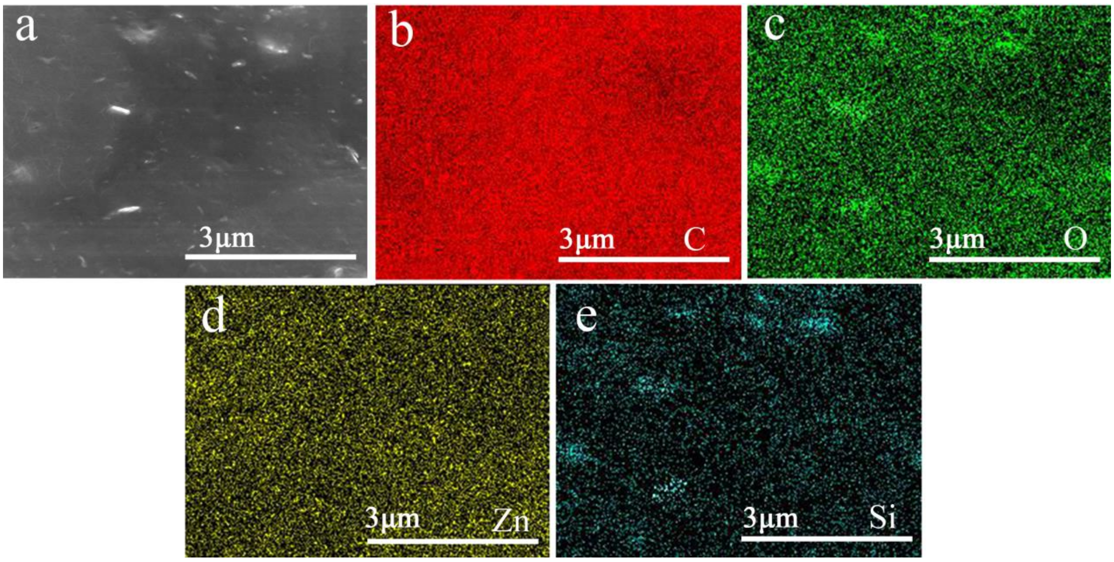

Figure S6 (a) SEM image and EDS maps of (b) C, (c) O (d) Zn , and (e) Si for in-situ-NC/ZDMA25

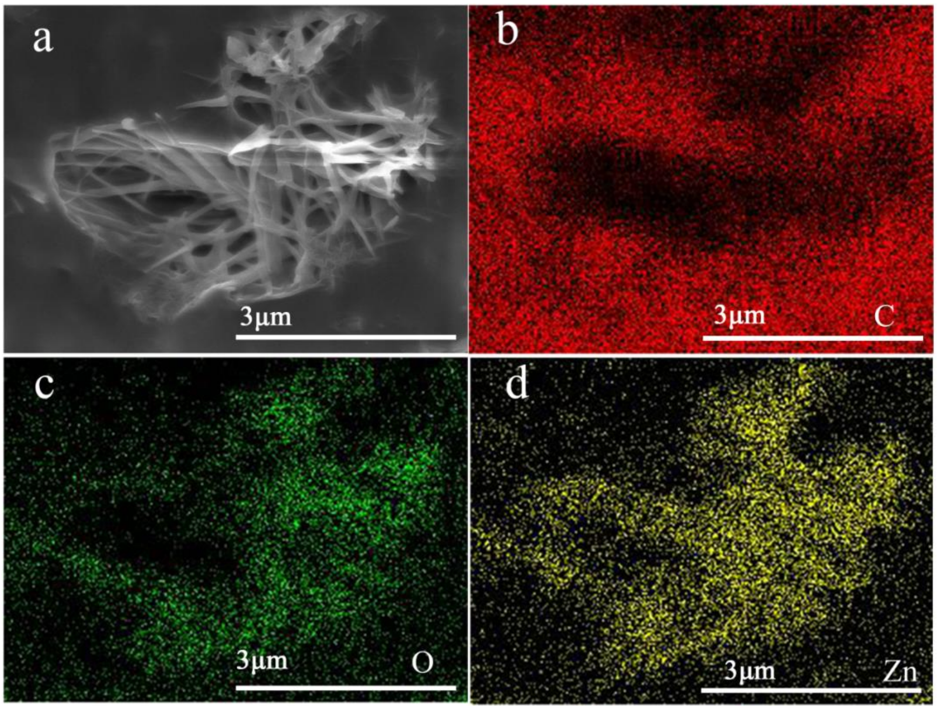

Figure S7 (a) SEM image and EDS maps of (b) C, (c) O, and (d) Zn for ph-NC/ZDMA40

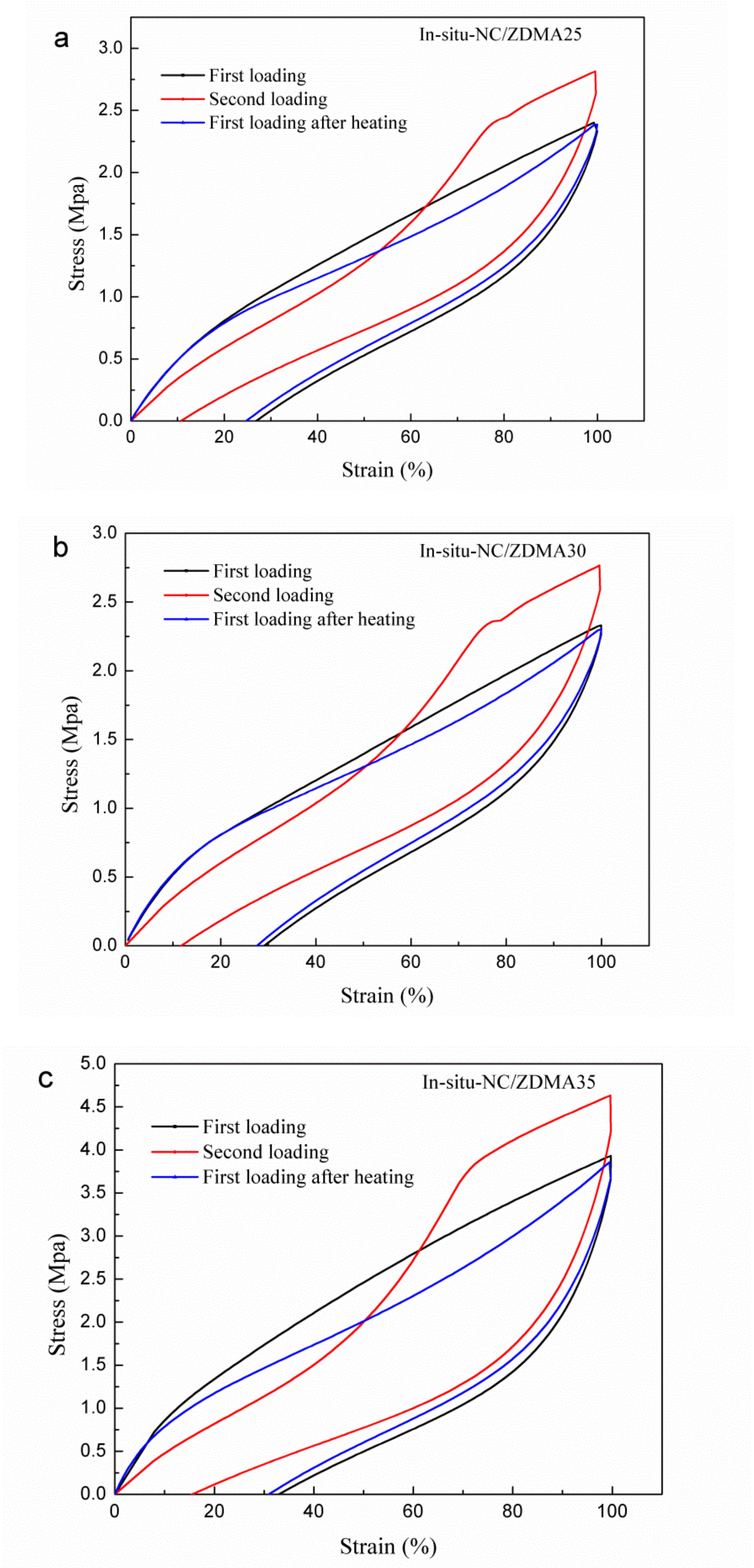

Figure S8 Cyclic tensile curves of (a) in-situ-NC/ZDMA25, (b) in-situ-NC/ZDMA30 and (c) in-situ-NC/ZDMA35

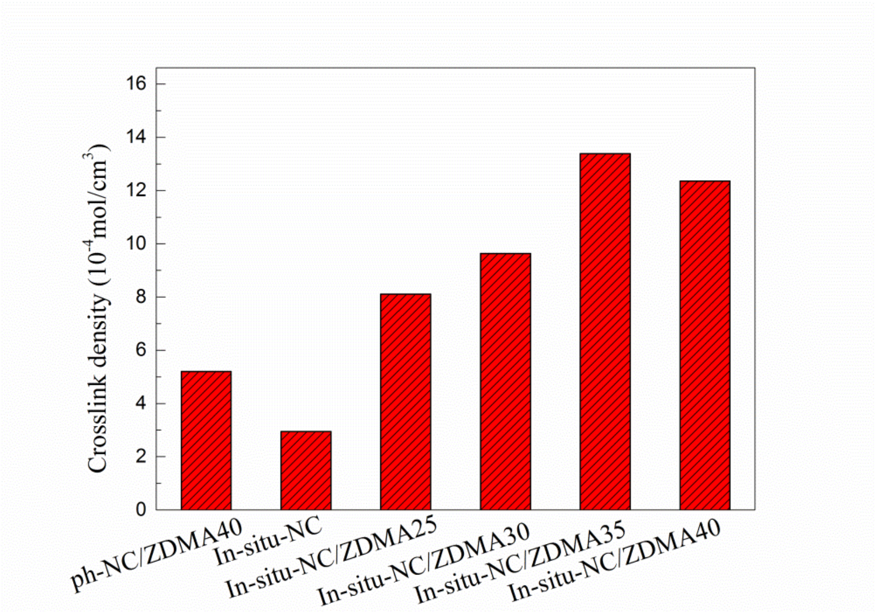

Figure S9 Total crosslink density calculated by DMA
